# Biomaterial Approaches to Enhancing Neurorestoration after Spinal Cord Injury: Strategies for Overcoming Inherent Biological Obstacles

**DOI:** 10.1155/2015/752572

**Published:** 2015-09-27

**Authors:** Justin R. Siebert, Amber M. Eade, Donna J. Osterhout

**Affiliations:** ^1^Lake Erie College of Osteopathic Medicine at Seton Hill, Greensburg, PA 15601, USA; ^2^Department of Cell and Developmental Biology, State University of New York Upstate Medical University, Syracuse, NY 13210, USA

## Abstract

While advances in technology and medicine have improved both longevity and quality of life in patients living with a spinal cord injury, restoration of full motor function is not often achieved. This is due to the failure of repair and regeneration of neuronal connections in the spinal cord after injury. In this review, the complicated nature of spinal cord injury is described, noting the numerous cellular and molecular events that occur in the central nervous system following a traumatic lesion. In short, postinjury tissue changes create a complex and dynamic environment that is highly inhibitory to the process of neural regeneration. Strategies for repair are outlined with a particular focus on the important role of biomaterials in designing a therapeutic treatment that can overcome this inhibitory environment. The importance of considering the inherent biological response of the central nervous system to both injury and subsequent therapeutic interventions is highlighted as a key consideration for all attempts at improving functional recovery.

## 1. Introduction

“One having a crushed vertebra in his neck; he is unconscious of his two arms (and) his two legs, (and) he is speechless. An ailment not to be treated” [1]. This excerpt from the Edwin Smith papyrus was the diagnosis of an ancient Egyptian physician, and some of the first ever medical observations regarding the limited ability of the central nervous system (CNS) to heal following a traumatic injury [1, 2]. This passage is also one of the first written accounts as to the grave nature of injuries to the CNS. While advances in modern medicine and technology have improved both lifespan and quality of life for victims of spinal cord injury (SCI), injury sustained to the spinal cord generally results in a permanent loss or impairment of motor function and sensation below the level of injury. This impairment presents victims of SCI with numerous financial, physical, emotional, and social burdens [3].

Current strategies to treat spinal cord injury have focused on restoring function via enhancement of neuronal survival after injury, regeneration of damaged axons, and neuroplasticity of spared axons. Ideally, a single treatment paradigm would be used to accomplish all of these tasks simultaneously. Unfortunately, however, research efforts have thus far demonstrated no single therapy or treatment that will reverse the damage after SCI. Such findings center on the fact that the spinal cord is a unique and complex environment, posing many challenges to the restoration of function. Given that combinations of pharmacologic and rehabilitative therapies may be necessary to address all of these challenges, researchers in this field need to consider the biological implications of each type of therapy in conjunction with the inherent response to spinal cord injury. Therefore, this paper is aimed at providing a comprehensive discussion of the challenges posed by the postinjury response of spinal cord, current strategies aimed at enhancing functional repair, and the potential use of biomaterials in aiding the recovery process.

## 2. Part I: The Complex Nature of Spinal Cord Injury

### 2.1. SCI: Etiology and Prognosis of Lesions

On average, there are approximately 12,500 newly reported cases of SCI in the United States each year, with a prevalence estimated to be approximately 276,000 persons [3]. Of the reported cases, a vast majority of SCIs (79%) occur in males and result from either a contusion or compression style injury [3]. During a contusion injury, forces are rapidly applied to and removed from the spinal cord. This causes a sudden and focal compression, with displacement of spinal tissue both rostrally and caudally, severing any axons within the affected region [4, 11, 12]. This style of injury is most commonly associated with blunt force trauma due to motor vehicle accidents (38%), falls (30%), and sporting injuries (9%) [3]. While compression injuries of the spinal cord occur as a result of a sustained force, crush injuries can result from slipped intervertebral discs, dislocation/fractures of the vertebrae, subluxation of the vertebrae during trauma, or spinal subdural hematomas and are known to produce larger and more diffuse areas of injury [3]. Although the area of injury can be quite extensive, trauma involving sharp penetrating injury or the complete dislocation of two adjacent vertebrae represents only a minority of SCI cases [3].

The human spinal cord, on average, is approximately 45 cm long in males and 42-43 cm long in females [13]. Trauma can occur anywhere along the length of the spinal cord. However, spinal injuries are largely localized to two anatomic regions: low cervical (C_5_-C_7_) and mid-thoracic (T_9_-T_10_, [3]). The anatomic level at which the injury occurs has a significant determination on the level and degree of impairment or paralysis that follows. Injuries to the cervical levels of the spinal cord most often result in some degree of quadriplegia, while those occurring at the thoracic region most often result in some degree of paraplegia [3].

While the anatomic level of the injury determines what regions and appendages of the body are affected, the completeness of the SCI determines the severity of loss in function and sensation. There are two categories into which SCIs can be classified: complete and incomplete. In a complete injury, the spinal cord is severed into two distinct stumps, axotomizing all of the ascending and descending axonal tracts. Incomplete injuries, on the other hand, axotomize some motor and sensory axonal tracts without separating the spinal cord into two distinct sections. The more complete the injury, the more severe the resulting impairment. In a complete lesion, the lack of tissue connectivity between the two cut ends results in physical retraction of the stumps, essentially negating any chance of translesion recovery. The spared rim of white matter in an incomplete lesion, however, holds the spinal cord together and provides a potential bridge for axonal regrowth, thus allowing for the possibility that some limited translesion recovery may occur. Unfortunately, even though a majority of SCI cases are the result of incomplete lesions [3], for which at least limited translesion recovery should be possible, the inherent biological response of the spinal cord to the injury often limits the success of the regenerative response. As such, characterizing the molecular features of the biological response, such that they may be addressed by therapeutic approaches, has largely been the target of SCI regenerative research.

### 2.2. Biological Response to Injury

#### 2.2.1. Neuronal Response to SCI

The basic anatomic organization of the spinal cord places the white matter tracts on the outer periphery, making them susceptible to physical trauma [13]. In considering contusive or crush style injuries, the physical stretching forces applied to the spinal cord appear to be concentrated at the nodes of Ranvier, which, being devoid of a myelin sheath, is the weakest point of the axon [4, 14]. Following axonal rupture, exposure of the axoplasm to the extracellular environment allows for the rapid influx of extracellular calcium, in turn activating phospholipase A_2_ and triggering the cut end of the axon to reseal [15, 16]. More importantly, the calcium dependent events that occur at the cut end following injury also appear to play a role in determining if the damaged axonal tip develops into an end bulb or a functional growth cone [17]. As the severed distal end of the axon undergoes the process of Wallerian degeneration, the proximal part of the neuron also undergoes a chromatolytic reaction [18]. This reaction is characterized by the movement of the nucleus to a more lateral eccentric position within the neuron's cell body and the rough endoplasmic reticulum taking on a fragmented appearance [18, 19].

The postinjury changes that occur in neurons are thought to be a result of a disruption in sustained neurotrophic support. As neurons rely on neurotrophins for survival, any alterations in the availability of such molecules could result in irreparable damage. In general, neurotrophic support can be provided through autocrine or paracrine sources or from axonal connections with a neuronal target [20]. The majority of neurotrophins arise from innervated targets of the neuron, at least during development [20, 21]. Following SCI, the termination of the link between the neuron and its target disrupts the continued supply of neurotrophic molecules, causing the neuron to atrophy and further decreasing its ability to mount a regenerative response [4, 19].

It is well known that supraspinal neurons lack a strong intrinsic regenerative response following an axotomy in the spinal cord. This class of neurons, which includes the corticospinal tract neurons (CST), vestibulospinal tract (VST) neurons, and rubrospinal tract (RuST) neurons, has, therefore, become the most frequent targets of neural regeneration research. Although the observed lack of a regenerative response fostered the belief that CNS neurons were incapable of undergoing any type of regeneration, some studies have demonstrated the contrary. It has been noted that CNS neurons, particularly those that were axotomized near the neuronal cell body, are able to grow axons within a peripheral nerve graft [22–24]. Similarly, propriospinal (PS) neurons, another class of CNS neuron intrinsic to the spinal cord, have also been demonstrated to grow into peripheral nerve grafts [25]. These studies suggest that if presented with the appropriate triggers and environmental conditions, CNS neurons can mount a regenerative response. However, it is also clear that the postinjury environment plays a significant role in quashing the regenerative capacity of CNS neurons.

#### 2.2.2. Glial Scar Formation

**Figure 1:**
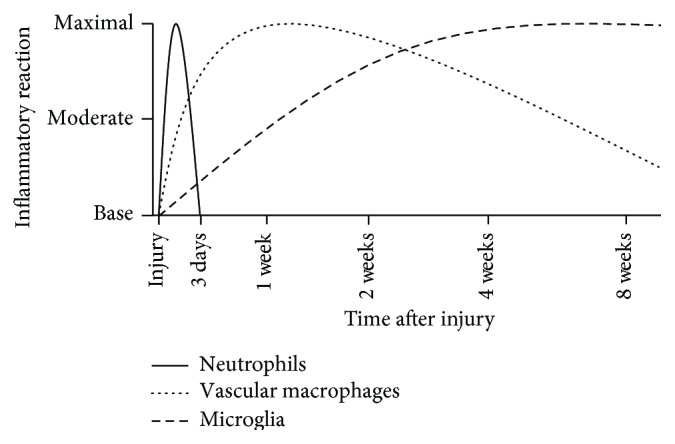
Immune response following SCI. Trauma to the spinal cord elicits an immune response, which begins almost immediately after injury. Neutrophils are the first immune cells to respond to the lesion site, arriving within the first few hours after injury, and remaining for up to 3 days after injury. Vascular macrophages are the second class of immune cell to arrive at the lesion, arriving after the initial infiltration of neutrophils. Activation and infiltration of vascular macrophages subsequently activate and recruit microglial cells, which can persist in the lesion site for months after injury [4–7].

The complex orchestration of molecular changes in the spinal cord following any trauma creates an environment that is well documented as hostile to the regenerative processes [4, 5, 26–31]. The physical forces applied to the spinal cord during a contusion injury result in the destruction of blood vessels, leading to a massive inflammatory response (Figure 1) and the creation of a hypoxic postinjury environment [6, 32–35]. This inflammatory reaction triggers the process of reactive astrogliosis, which leads to the production of a chemophysical barrier that inhibits regenerative activity [4, 5, 26–28, 36–38]. More specifically, immediately following an SCI, astrocytes located within the zone of injury become hypertrophic, extending their processes, proliferating, and organizing into a dense astrocytic rich border at the lesion site [5, 26, 29]. Overall, this process functions to produce a glial scar surrounding the lesioned area.

The glial scar has been widely accepted as a primary reason for the lack of a maintained regenerative response following SCI. However recent evidence is beginning to cast a new light on the glial scar as an important protective barrier preventing further secondary tissue damage [28, 39–41]. These studies also suggest that the formation of the glial scar may be beneficial for the initiation of the axonal sprouting response. For example, Faulkner and colleagues [39] demonstrated that ablation of reactive astrocytes following a stab or crush style injury to the mouse spinal cord effectively prevented formation of the glial scar. This process resulted in increased demyelination and degeneration of neurons but decreased number of oligodendrocytes and functional ability. Overall, reactive astrocytes have been discussed as benevolent in nature, at least to some degree, due to their ability to reduce the excitotoxic levels of glutamate in the extracellular environment, produce molecules that prevent oxidative damage and toxicity, allow for reformation of the blood brain barrier, and regulate the fluid and ion balance of the extracellular space [28].

#### 2.2.3. Chondroitin Sulfate Proteoglycans (CSPGs) as Barriers to Repair

**Figure 2:**
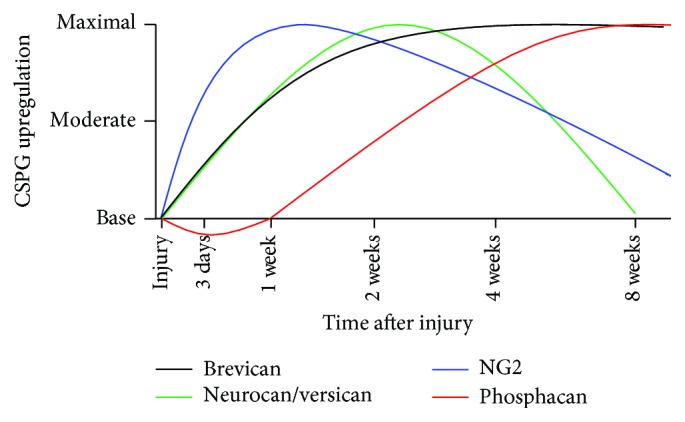
Upregulation and expression of CSPGs. Almost immediately following an SCI, astrocytes located within the area of trauma begin to undergo hypertrophy, synthesizing and secreting CSPGs, including neurocan, phosphacan, and brevican. Additionally, the infiltration of vascular macrophages, activated microglial cells, and OPCs results in the increase in the proteoglycans NG2 and versican. The temporal expression of these proteoglycans is important to factor into any treatment, as they have differential effects on the regenerative process. Neurocan and versican are upregulated quickly following injury, with maximal expression observed 2 weeks after injury. Their expression begins to wane at longer times, approaching base levels by 8 weeks after injury. Brevican is also upregulated after injury, reaching maximal expression 2 weeks after injury. However unlike neurocan and versican, brevican expression remains elevated over time. Phosphacan is initially downregulated following SCI, with significantly reduced levels 1 week after injury. The expression begins to increase at longer times after injury and peaks around 8 weeks after injury. NG2 expression can be generally correlated to the infiltration of vascular macrophages, activated microglia, and OPCs, with maximal expression being found 1 week after injury. This differential expression pattern of CSPGs plays a large role in governing the regenerative response as many CSPGs are inhibitory to both process of atonal regeneration and remyelination (adapted from [8]).

Reactive astrogliosis produces an upregulation of CSPGs in the tissue surrounding the lesion site (reviewed by [5, 37, 38, 42–45]). Immediately following injury, astrocytes upregulate and synthesize the CSPGs brevican, neurocan, and phosphacan, while infiltrating vascular macrophages and microglia increase their expression of the proteoglycan NG2 (Figure 2, [8, 43, 46]). Demyelination triggers the recruitment of oligodendrocyte progenitor cells (OPCs) to the lesion site, which also causes the increase in expression of the proteoglycans NG2 and versican [8, 43, 46]. High levels of CSPGs in the postlesion environment have a significant role in inhibiting the regenerative capabilities of the CNS. For example, in the presence of CSPGs, OPCs fail to undergo differentiation into myelin forming oligodendrocytes [47–49]. The interaction of neurons with CSPGs activates the Rho-ROCK and/or protein kinase C (PKC) inhibitory signaling cascades, which have been demonstrated to negatively regulate axonal outgrowth and extension by inducing growth cone collapse [50, 51]. This leads to growth cone retraction and an overall abortion of the regenerative process [4, 5, 36]. Blocking the Rho-Rock and/or PKC pathways has been noted to reverse the inhibitory effects of CSPGs on axonal regeneration and OPC process outgrowth [45, 47, 52, 53], adding additional confirmation for the effects of these signaling cascades.

While CSPGs, overall, exert a largely inhibitory influence to the regenerative process, the specific inhibitory nature varies among the different proteoglycans.* In vitro*, purified brevican, neurocan, and phosphacan have all been identified as inhibitory to axonal attachment and growth [5, 42]. The ability of neurocan and phosphacan to interact with neural cell adhesion molecules (N-CAM) on neurons is thought to be the mechanism underlying their inhibitory effects [5, 42]. Versican, however, is not inhibitory to either axonal regrowth or adhesion. This is evidenced by the* in vitro* finding that axons not only are able to grow through deposits of versican but also show no signs of inhibition in the presence of the purified proteoglycan [54, 55]. Some* in vitro* studies have demonstrated that neural/glial antigen 2 (NG2) is inhibitory to the process of axonal outgrowth, although the effects of NG2* in vivo* remain undetermined [8, 56, 57]. The most recently characterized brain-derived proteoglycan, Te38, has been found to be highly inhibitory to axonal regeneration [58] and is readily present within the lesion site following SCI [59]. While Te38 is able to be detected for up to 4 weeks after injury, the exact expression pattern for this proteoglycan has yet to be determined [59].

Neurocan and phosphacan are also both highly inhibitory to OPC process outgrowth and differentiation [47]. Importantly, studies have shown that CSPGs interact with adhesion molecules expressed on various cell types, mediating their inhibitory effects via the surface receptor protein tyrosine phosphatase sigma (PTP*σ*) that is found on both neurons and OPCs [49, 60–63]. This is an important finding since, to date, all other CSPG receptors, including Nogo-66 Receptor 1 (NgR1), Nogo-66 Receptor 3 (NgR3), and leukocyte common antigen receptor (LAR), have only been found on neurons [64, 65].

Overall, the inhibitory influence of CSPGs on the postinjury environment is a major barrier to the regenerative process. Further complicating this matter is the temporal expression of these molecules. While the induction of the CSPG synthesis begins immediately after injury, the upregulation of specific CSPGs happens at different intervals. Brevican, neurocan, and versican expression is found to be maximal at two weeks after injury, while NG2 achieves peak expression one-week after injury (Figure 2, [8]). Interestingly, however, the expression of phosphacan is initially downregulated and then begins to be expressed, with peak levels found approximately eight weeks after injury [8]. The continual upregulation of different CSPGs makes the postinjury environment of the spinal cord inhibitory to the regenerative process for many months.

The glial scar appears to be a paradoxical structure, identified as highly inhibitory to axonal regeneration, while also protecting and isolating the damaged tissue. The dual nature of the glial scar suggests that while it will need to be modified in order to create a permissive environment, the scar is necessary to prevent additional tissue damage. Additionally, the fact that reactive astrogliosis is a response that is graded to the nature of the CNS insult [29] indicates that treatments targeting this process will bring the most benefit to individuals suffering from severe injury.

#### 2.2.4. Myelin Degradation

Both direct physical destruction and indirect damage due to inflammatory activity result in the death of oligodendrocytes, the myelin producing cell of the CNS. Oligodendrocytes are particularly sensitive to SCI [66] with tissue damage after an SCI resulting in the death of oligodendrocytes at the lesion site and, over time, even at a distance from the initial lesion [5]. Oligodendrocyte death occurs in two stages, with the initial loss being due to physical damage during the injury process and a delayed secondary loss resulting from ongoing pathology [4, 5, 7]. While the majority of early oligodendrocyte death is necrotic [7], oligodendrocyte apoptosis can be observed, both locally and in segments at a distance from the site of original lesion, for weeks following injury [67–74]. It has been demonstrated that a compression injury inflicted at the T8-9 level can lead to oligodendrocyte apoptosis at spinal levels as far away as T1-L2 [68]. This secondary process occurs, in part, due to SCI induced glutamate release, which reaches levels that are toxic to oligodendrocytes (550 mM ± 80 mM, [75]). Other events that induce apoptosis are the formation of free radicals in the lesioned tissue [7] and p75 neurotrophic receptor (p75^NTR^) mediated cell death. The latter results from p75^NTR^ upregulation following pathologic stress to the oligodendrocytes [76, 77]. Specifically, trauma to the spinal cord leads to increases in the synthesis and production of nerve growth factor (NGF) by astrocytes, activated microglia, and vascular macrophages [78]. The immature form of NGF (proNGF) interacts with p75^NTR^ on the surface of oligodendrocytes, resulting in apoptosis [79].

Loss of oligodendrocytes creates an excess of myelin breakdown products in the lesion. This myelin debris contains variety of myelin proteins, including Myelin Associated Glycoprotein (MAG), Myelin Oligodendrocyte Glycoprotein (MOG), Nogo-66, and Nogo-A, all of which have been demonstrated to be highly inhibitory to regenerating neurons [63, 80–90]. These molecules interact with a variety of surface receptors, such as Nogo receptor (NgR aka NgR1), p75^NTR^, and TROY (aka TAJ), and are documented to provide repulsive axonal guidance cues, by collapsing or causing retraction of the axonal growth cone [5, 44, 84, 89, 91, 92]. Due to the slow phagocytic nature of the central nervous systems macrophages and microglia, myelin proteins are able to remain in the postinjury environment for several months [5, 32, 93]. These proteins, however, are not the only inhibitory elements found at the lesion site. The regenerative process is also inhibited by the presence of glycoprotein CD44, tenascins, and semaphorins at the site of injury (reviewed by [4, 5, 26]).

#### 2.2.5. Cavitation and Cyst Formation

As the macrophages and microglia clear cellular debris, the lesion cavity will eventually become nothing more than a fluid filled cyst called a syrinx [4, 7, 94]. While the pathophysiology underlying the formation of the posttraumatic syrinx is not fully understood, the process of postinjury cavitation is observed in both human and rodent cases of SCI [94, 95]. In the rat model of spinal cord injury, cavity formation is usually observed 15 days after injury [95]. Mouse models of SCI, interestingly, do not typically demonstrate such postinjury cavitation [95]. Syrinx formation in humans after SCI can take up to several months or years to manifest [94]. These cysts, once formed, run the risk of enlarging, producing a degenerative condition known as syringomyelia, which has the potential to cause further deterioration of sensory or motor function [94]. This fluid filled cavity also presents the surviving neurons, yet another challenge to overcome in the regenerative process, by eliminating the availability of an extracellular matrix on which their axons can grow.

Given the substantial role that cellular and molecular responses play in the regenerative ability of the spinal cord, it is critical that these aspects are considered when attempting to successfully approach the development and use of methods aimed at neurorestoration following SCI. Therefore, the remainder of review will be devoted to discussion of common research strategies for enhancing repair as well as the potential role of biomaterials in promoting more substantial neurorestorative effects.

## 3. Part II: Current Research Strategies to Enhance Repair

Given the complexity of the biological response to SCI, a number of different therapeutic approaches have been developed to target one or more of the issues preventing functional recovery. In general, spinal cord injury research focuses on a few broad topics: neutralization of inhibitory elements within the postinjury environment; promotion of neuronal survival (neuroprotection); stimulation of axonal regeneration and/or plasticity (neuroregeneration); and remyelination of denuded axons. Research in each one of these areas has yielded important insight into the ability for neurorestoration of the functional spinal cord as a result of postinjury environment manipulation.

### 3.1. Neutralization of Inhibitory Factors

Neutralization of inhibitory factors in the postinjury environment is one promising approach for enhancing the regenerative response following an SCI. While there are many different inhibitory elements that can be targeted within the postinjury environment, the most progress has been made on developing agents to neutralize the inhibitory influence of either CSPGs or myelin debris.

CSPGs expressed in and around the glial scar are widely accepted as a primary reason for the lack of axonal regeneration and/or remyelination following an SCI. However, the inhibitory nature of the CSPGs can actually be neutralized using the enzyme chondroitinase ABC (cABC). Chondroitinase is an enzyme produced by the bacteria* Proteus vulgaris*, which catalyzes the removal of the glycosaminoglycan side chains from the central core protein [96]. Many studies have shown that by treating a CNS lesion site with the enzyme cABC both axonal sprouting and axonal growth into and around the lesion are significantly increased [97–103]. The use of this agent has been shown to effectively reverse CSPG inhibition and promote axonal sprouting and outgrowth [97–102, 104]. Use of cABC also enables the migration and differentiation of endogenous OPCs [47–49, 105].

One of the major limitations of cABC as a treatment for SCI is the mode of administration. Chondroitinase is a very labile enzyme that when reconstituted does not retain its biological activity for very long, due to its thermal instability. When incubated at 37°C, the enzymatic activity of cABC, in solution, is gone by 7–10 days [106]. While the therapeutic ability of cABC is very promising, experimental stabilization of the enzyme is needed for it to be a more effective therapeutic agent. Stabilization of cABC has been attempted in several ways including alteration of its structure [107], incorporation in viral vectors for constant* in vivo* expression [103, 108, 109], or incorporation into biomaterial delivery systems [110].

While the neutralization of CSPGs has demonstrated promise in reversing their inhibitory effects, a similar result has also been noted via modulation of the activity of the PTP*σ* receptor. The PTP*σ* receptor has been identified as a CSPG interacting receptor that is expressed on neurons [49, 60–63]. In general, PTP receptors are a group of surface receptors that contain two catalytic domains, D1 and D2. The D1 domain is the primary catalytic site, while D2 serves regulatory functions. One way in which the activity of these receptors is modulated is through a wedge shaped sequence that is located between the membrane and the proximal region of the D1 catalytic domain [111]. Therefore, the activity of the PTP*σ* receptor can be inhibited using a generated membrane-permeable peptide that mimics the PTP*σ* wedge sequence. The binding of this peptide to the PTP*σ* receptor has been noted to result in a significant increase in axonal growth after injury [112]. As a result, research has determined that systemic delivery of this peptide over time allows for both the enhancement of serotonergic innervation of the spinal cord below the level of injury and the facilitation of recovery of motor function and micturition in treated animals [112].

The myelin debris released into the lesion environment presents additional inhibition to the regenerative ability of the injured axons, which becomes further compounded by the slow phagocytic clearance of the debris [5, 113, 114]. One myelin associated protein known to be a potent inhibitor of axonal regeneration, Nogo-A [115], is a specific target in the quest to neutralize inhibitory factors. Just as the inhibitory CSPGs can be neutralized using cABC or by modulating CSPG receptors with a blocking peptide, the negative influence of Nogo-A can be ameliorated using antibodies against it.

Significant increases in both the number of axons regenerating and the overall length of the regenerating axons have been found following infusion or other systemic deliveries of the Nogo-A antibody [85–88, 90]. Even after a long interval of time following a stroke injury, treatment with anti-Nogo-A can still produce not only a sprouting response from the damaged axons but also an improvement in subsequent functional recovery [116]. While the effects of Nogo-A on axonal regeneration and sprouting have largely been studied utilizing the stroke model of injury, its use in SCI models also demonstrates axonal sprouting and increases in the length of axonal arbors [87]. The observed neurite outgrowth, attained by using antibodies to Nogo-A, is thought to be accomplished through the inhibition of intracellular pathways that are activated by the Nogo receptor, such as the Rho/Rock pathway [52, 117].

Similar effects on axonal regeneration have also been noted following administration of an antibody that is specific to the potent inhibitory domain of Nogo-A, IN-1 [118, 119]. Notably, neuroanatomical evidence demonstrating regenerative axonal growth in combination with marked improvements in the recovery of function has been found when the IN-1 antibody is delivered to the site of a cerebral cortical transection or stroke injury in mice [118, 119]. More importantly, when IN-1 is administered into the CNS of nonhuman primates following a thoracic SCI, significant increases in axonal sprouting and regenerative growth are also found [120].

Taken together, these studies collectively demonstrate that the use of cABC, Nogo-A, or IN-1 to neutralize the inhibitory elements found within the postinjury environment has potential to aid the neuroregenerative response following SCI.

### 3.2. Stimulation of Axonal Regeneration

CSPGs interfere with axonal regeneration by inducing collapse of axonal growth cones, producing premature abortion of the normal regenerative response. Axonal collapse is thought to be a result of molecular signaling events activated within the axon itself. Exposure of the damaged axonal tip to the CSPGs and myelin debris found within the lesion results in the activation of inhibitory signaling pathways, such as RhoA/Rock. This then triggers the breakdown of actin filaments and results in the cessation of axon growth [121]. As stability of the axonal growth cone is dependent on microtubule polymerization, which is regulated by microtubule-actin interactions, a recent avenue of research has focused on promoting axon regeneration via microtubule stabilization and/or the modification of axonal pathway signaling.

Microtubule stabilizing anticancer drugs, which achieve their anticancer properties by interfering with cellular division, have recently shown promise in the field of axonal regeneration. Two such drugs are paclitaxel (Taxol) and Epothilone B [121–123]. Taxol has been found, both* in vitro* and* in vivo*, to prevent the formation of retraction bulbs after injury, stabilize the cytoskeleton of the reactive growth cone, and promote the regeneration of axons in an injured optic nerve model [121, 123]. Epothilone B, when given systemically following an SCI in rodents, has been found to decrease glial scarring and increase microtubule polymerization in the tip of the axon. In short, induction of microtubule polarization in the growth cone appears to drive growth of the axon at the site of lesion [122]. Importantly, not only do both Taxol and Epothilone B have the potential to enhance axonal growth following injury but also both of these drugs are currently FDA approved for cancer therapy. Thus, some evidence related to a degree of safety for use of such drugs in humans has been previously established in the cancer literature.

Axonal regeneration may be stimulated after injury through direct modulation of signaling pathways within the axons. While the molecular signaling events that occur within the axon are complex and numerous, there are a few that warrant discussion due to their ability to facilitate axonal regrowth. One such molecular signaling target is Phosphate and Tensin homologue (PTEN), which is a negative regulator of the mammalian target of rapamycin (mTOR). Recent studies have demonstrated that silencing this molecule results in significant axonal growth [124–126]. The disruption of PTEN, via mouse knockout models, has also been noted to produce robust axonal regeneration following a crush injury to the optic nerve [124, 125]. Even injecting shRNA against PTEN prior to injury appears to allow for protection of the regenerative response. Injections of shRNA against PTEN into the CST neuronal cell bodies of neonatal mice have been found to produce significantly higher levels of postinjury axonal regeneration, as compared to controls, when spinal cord crush injury had occurred 7 weeks after administration of the injection [126]. The regeneration that occurred following the shRNA injections was even present across areas rich in GFAP.

In addition to PTEN, suppressor of cytokine signaling 3 (SOCS3), which is a negative regulator of Janus kinase/signal transducers and activators of transcription (JAK/STAT), has been described as inhibitory to axonal regeneration. For example, conditionally knocking out SOCS3 in mice results in a significant increase in the number of axons that cross a crush injury to the optic nerve [127]. Further, when both SOCS3 and PTEN are knocked out, the amount of axonal regeneration observed following an optic nerve crush injury is significantly greater than what is achieved by knocking out only PTEN or SOCS3 alone [125].

Finally, another target for axonal regeneration therapies is the Krüppel-like factors (KLF) family of transcription factors. This family of transcription factors plays a large and important role in the regulation of neural growth and regeneration by either suppressing or enhancing axonal growth abilities. Interestingly, KLF family members known to be inhibitory to axonal growth (KLF 4 and 9) have been found to be upregulated postnatally, while those that are growth promoting (KLF 6 and 7) are downregulated at this time [128, 129]. Although this may sound counterintuitive to axon growth, which occurs at a high rate during development, knowledge of the levels of expression during times of high development provides another avenue for research into mechanisms for targeting regenerative potential.

These studies, when considered collectively, indicate that both strategies that target growth inhibitory signaling elements and therapies that stabilize the growth cone may be necessary in order to achieve the long distance growth needed for functional recovery following SCI.

### 3.3. Neurotrophic Factor Supplementation

The inhibitory nature of the postinjury environment is well described, and the physiologic and metabolic stresses experienced by the neuron are extensive. While it is clear that the neutralization of inhibitory elements found within the post-SCI environment has beneficial effects on axonal sprouting/growth, the overall health of neurons following injury still needs to be maintained. If the neuron dies, then any hope of a regenerative response is lost. Therefore, another active research area in SCI regeneration has focused specifically on the neuron, identifying ways to promote neuronal survival, axonal regeneration, and axonal plasticity through the use of neurotrophic (NT) agents and other growth promoting molecules.

Neurotrophic molecules consist of a family of proteins which are structurally similar and bind to one of three tyrosine kinase (Trk) surface receptors or the p75 neurotrophic receptor (p75^NTR^). Members of the NT family include Brain-Derived Neurotrophic Factor (BDNF) and neurotrophin-4 (NT4/5) which preferentially bind to TrkB, NGF which binds TrkA, neurotrophic factor-3 (NT-3), and its receptor TrkC [130–132]. While NTs bind to a specific Trk receptor, all of the NT molecules can bind to p75^NTR^, which has important physiological implications on neurons. A NT binding to p75^NTR^ only without the expression of the appropriate Trk receptors can be harmful. For example, when sympathetic neurons expressing p75 and TrkA receptors were exposed to BDNF, the binding of BDNF to p75^NTR^ without the presence of TrkB resulted in p75^NTR^ induced apoptosis of the neurons [131–133].

Another family of growth promoting molecules is the glial derived neurotrophic factors, which require two surface receptor components. The GDNF family of molecules directly binds to one of four GDNF family receptor alphas (GFR*α*), which then complex with the Ret receptor tyrosine kinase. Members of this family include glial derived neurotrophic factor (GDNF) which binds GFR*α*1, neurturin which binds GFR*α*2, artemin which binds GFR*α*3, and persephin which binds to GFR*α*4 [132, 134].

The NT and GDNF family of molecules represents only a small sample of the plethora of neurotrophic substances and growth factors that may contribute to the regenerative quality of the CNS. Additional neurotrophic agents, which have also been shown to be potent in enhancing neuronal survival or axonal regeneration, include leukemia inhibitory factor (LIF) and ciliary neurotrophic factor (CNTF) [135–138]. Importantly, each of these additional agents binds to specific surface receptors, whose location and binding affinity must be considered when investigating their potential to assist in the regenerative process. Through the use of* in situ* hybridization, immunofluorescence, and genetic screening techniques, Trk, Ret, and GFR*α* receptors have been localized in several classes of afferent neurons, efferent neurons, and interneurons (e.g., [139–143]). If a specific neuronal population expresses a certain class of neurotrophin receptors, the neurons are considered to be responsive to that neurotrophin. Thus, BDNF, GDNF, NGF, NT-3, and NT-4/5 are commonly utilized in attempts to prevent injured neurons from undergoing apoptosis. Moreover, these factors have been used in order to coax such neurons into a regenerative response.

Studies have demonstrated that classes of efferent neurons such as CST, RuST, and coerulospinal and reticulospinal neurons are receptive to the NT agents, BDNF, GDNF, NT-3, and NT-4/5, with varying responses of neuronal survival, axonal sprouting, and even axonal growth [141, 144–148]. BDNF and NT-4/5 treatment has been shown to prevent the atrophy of RuST neurons, stimulate an upregulation of genes known to be associated with axonal regeneration, and even promote the regeneration of RuST axons [146, 147]. CST neurons have shown resistance to postinjury apoptosis following BDNF treatment [20] and have also been shown to be protected from cell death by the neurotrophic factors GDNF and NT-3 [141, 145, 148]. NT-4/5 promotes the growth of reticulospinal, coerulospinal, and PS axons, while appearing to have no significant effect on other efferent classes of neurons, such as the CST neurons [149]. While BDNF, NT-4/5, and GDNF are effective in coaxing classes of efferent neurons to undergo some degree of post-SCI sprouting, afferent classes of neurons, such as the dorsal root ganglion cells, are responsive to NT-3 and NGF [150, 151]. When considering or utilizing NT agents as a therapeutic strategy, there are three major issues that need to be carefully examined: the location of NT administration, the timing after injury in which the NT agent should be administered, and finally the number or combination of NT agents that need to be or can be administered at one time.

Overall,* in vitro* studies have shown that supplying neurotrophins, at the cell body and axon terminals combined or simply at the axon terminals alone, can maintain the neuronal cell body and induce axon growth [152]. In some cases where neurotrophic treatment is only applied to the cell body, axons are found to retract despite survival of the neuron [152]. However,* in vivo* studies of CST neurons demonstrate that BDNF treatment of the CST neuron at the cell body following axotomy saves the neuron from cell death and promotes sprouting of the injured axon [20, 153, 154], while damaged CST axons show no signs of regenerative growth when BDNF is applied at the lesion site in the spinal cord [20]. Treatment of RuST neurons with the neurotrophic factors at the level of the brain not only prevents their atrophy but also has been found to promote their axonal regeneration [146, 147]. Deciding on which location to apply the NT treatment is complicated, while the damaged end of the axons in cases of SCI is easily accessible, and the cell bodies of the major efferent neuronal classes are located in the brain and brainstem. This raises the question of whether or not performing invasive brain surgery to gain access to the neuroanatomical locations of the efferent neurons and potentially causing tissue damage via the administration of a NT agent are worth the risk posed to the patient.

**Figure 3:**
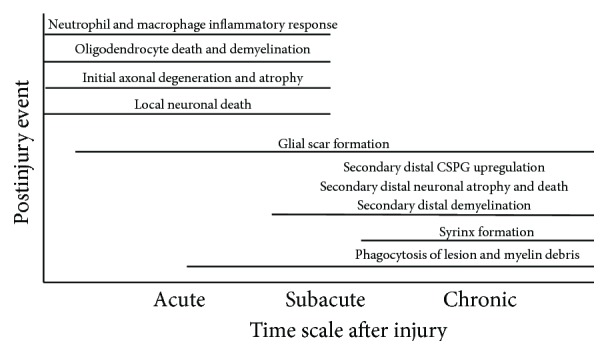
Chronology of postinjury events. The lesion site in an injured spinal cord is also a very dynamic environment that undergoes many different changes, as the lesion changes from an acute injury to a chronic injury. In addition to the inhibitory environment established after SCI, the ever-changing nature of these postinjury events needs to be factored into the design of any therapeutic treatment [4, 5, 7, 9, 10].

Timing of NT delivery is another critical aspect when it comes to the use of NT agents to promote repair in the lesioned spinal cord. The lesion site and surrounding tissue present a very dynamic environment, with a multitude of events occurring concurrently (Figure 3). This complex orchestration of events (see review [9]) shifts from an acute phase of inflammation and tissue necrosis, resulting in an immediate loss of neurons and myelin, to a chronic phase of secondary injury, where the CNS structures distal to the site of injury undergo neuronal atrophy, CSPG upregulation, and demyelination (Figure 3). Neuronal response to NTs can be significantly impacted by the time of administration. In rodent models of SCI, BDNF and/or NT-3 delivered either immediately or 7 days after transection has been found to produce differential effects on axonal regeneration and functional recovery. Specifically, significantly greater amounts of axonal regeneration and behavioral recovery were observed in animals that received the delayed NT treatment, as compared to those having received treatment immediately following the spinal transection [155]. In contrast, immediate treatment of injured RuST neurons with BDNF results in robust growth of damaged axons in the spinal white matter [156]. However, when BDNF is provided at the lesion site several days following an injury, it appears to have no effect on RuST neurons [142]. The response of RuST neurons to BDNF illustrates another critical aspect of the issue of timing; traumatic injury to CNS neurons can cause a differential and transient expression of surface receptors that bind to specific neurotrophins. Examples of this postinjury shift in expression were observed in TrkB surface receptor on RuST neurons [142]. While TrkB is expressed along the entire axon and cell body of uninjured RuST neurons, the axonal expression of TrkB following a traumatic injury was found to diminish as the interval after injury increased [142, 147]. At 1 and 2 months after injury, TrkB receptor expression is found to be localized only to the RuST cell body with no expression on the reactive ending of the injured axon [142, 147]. This finding offers an explanation as to why BDNF treatment at the RuST cell body is successful in promoting growth and survival as well as axonal sprouting, while treatment at the damaged reactive ending appears to have no effect on the RuST neurons [142, 147].

Microarray studies examining the postinjury response of specific classes of CNS neurons have also demonstrated how critical the issue of timing is in regard to the regenerative response. In a study examining the response of short thoracic propriospinal (TPS) neurons to axotomy, a strong upregulation in the genes for the receptors of GDNF and LIF was observed 3 days after injury [143]. Even more interesting was the upregulation in the NT receptor genes that occurred concurrently with the upregulation in several genes commonly associated with axonal regeneration. Following the 3 days after injury, gene expression level for both NT receptors and regeneration-associated genes began to decrease [143]. Therefore, the timing of NT administration may need to be specifically tailored to the postinjury expression curve of the NT receptors for individual populations of neurons in order to maximize the regenerative potential of these cells. Further, another important aspect of NT treatment is the timeframe in which the NT agents will be needed in the postinjury environment. Axonal growth proceeds at a very slow rate. Therefore, NT therapy will need to be administered in a manner that will allow the agent to be present in the lesion site for many months after injury.

Given the sheer number NT and growth factor receptors that are expressed on neurons and the differential expression of these receptors in efferent and afferent neuron populations, it is likely that different combinations of these NT molecules will be needed in order to elicit full regenerative potential after injury. To this end, studies have shown enhanced regenerative responses in retinal ganglion cells following application of a combination of BDNF, CNTF, fibroblast growth factor (FGF2), and NT-3, as compared with use of each factor independently [157–159]. In the study examining the postinjury effects of TPS neurons, examination of PS neurons after injury showed an upregulation in the receptors for GDNF and LIF, with no change in expression for the BDNF, NT-4/5, and NT-3 receptors [143]. The expression of many different NT receptors in TPS neurons strongly suggests that multiple agents (BDNF, NT-3, NT4/5, GDNF, and LIF) may be necessary for a strong and sustained regenerative response.

Administering neurotrophic agents after SCI results in an increase in the percentage of neurons spared from atrophy and apoptosis, as well as an enhancement of axonal sprouting or regeneration, when compared to control groups [20, 147, 156, 160]. It is clear however that many questions and problems with NT supplementation still exist. While considering which NT agents to give and how best to provide them simultaneously, the response of the other cell populations, glial cells, and immune cells, to each individual NT administered will have to be addressed. In addition to the critical issues of location and timing noted above, another important limitation that will need to be overcome is the formation of the “sink” or “honeypot” effect [161]. This occurs when the injection or infusion site has such a high concentration of NT agent and the sprouting/regenerating axons or migrating OPCs do not move beyond or outside of borders of this location [159, 161]. While NT administration will definitely have to be part of any postinjury regenerative therapy, there are still many issues that need to be resolved.

### 3.4. Remyelination

An additional avenue that can enhance functional recovery after SCI is the process of remyelination. As previously discussed, the survival of myelin producing oligodendrocytes can be limited by both direct and indirect factors following SCI. While the exact axonal cues that mediate oligodendrocyte survival have not been fully elucidated, both* in vivo* and* in vitro* experiments have demonstrated that the degeneration of axons subsequently results in the death and degeneration of oligodendrocytes (see review [162]). Further, when oligodendrocytes undergo apoptosis, all axons that are wrapped by that particular oligodendrocyte undergo the process of demyelination. Without myelin, the saltatory conduction of action potentials across the demyelinated portions of the intact axons can be severely impaired, exacerbating the postinjury deterioration of function [4, 5]. This process may be a critical component of the limited potential for functional recovery, given that one oligodendrocyte can be responsible for myelinating up to 60 different axons [163].

Remyelination of axons depends upon the health and availability of OPCs. Upon the completion of initial axon myelination, populations of adult OPCs remain throughout the brain and spinal cord. In order for OPCs to be successful in remyelinating axons, they must be able to proliferate, migrate towards the site of demyelination, make contact with an axon, and then mature into myelin forming oligodendrocytes [5]. These remaining progenitor cells are highly responsive to a demyelinating lesion (see reviews [164–166]), with those located within about 2 mm of the lesion site appearing to migrate towards the site of demyelination [167]. Unfortunately, however, the postinjury environment created following an SCI is highly inhibitory to the OPC, preventing both migration and development of these cells [47–49]. Additionally, the infiltration of OPCs into the lesion site and subsequent remyelination of denuded axons are minimal to nonexistent as the OPCs only accumulate at the border of the lesion [105, 165, 166]. CSPGs expressed in the glial scar are also highly inhibitory to the process outgrowth and differentiation of OPCs [5, 47–49, 63, 165, 168]. While this inhibitory influence is thought to be a major reason for failure of spared axon remyelination, an alternative theory posits that mature and damaged axons are no longer able to undergo the process of myelination.

The hypothesis that adult axons are no longer capable of myelination has been addressed in a series of different studies. In the adult retina, the nerve fiber layer contains naturally unmyelinated axons, as OPCs are unable to migrate out of the optic nerve and myelinate these axons. However, when OPCs are transplanted into the nerve fiber layer and then the layer is examined 4 weeks after OPC transplantation, axons have been found to undergo myelination [169]. The ability of demyelinated axons to remyelinate has also been demonstrated utilizing the cuprizone model of demyelination. Although cuprizone, when added to the diet of lab animals, results in demyelination, it has been demonstrated that spontaneous remyelination occurs quickly after removal of this drug from the animal's diet [170–173]. Collectively, these studies argue against the hypothesis that adult or demyelinated axons are no longer capable of undergoing (re)myelination and that the one likely cause of remyelination failure after CNS injury is the formation of the glial scar.

Interestingly, experimental therapies commonly used to stimulate axonal sprouting and regeneration after SCI have also demonstrated effects on the biology of OPCs. Supplementing the lesion site with various neurotrophic factors such as BDNF, NT-3 [174], or other growth promoting agents, that is, apotransferrin [175] resulted in enhanced levels of remyelination after injury. It has also been demonstrated* in vitro* that when BDNF, NT-3, and GDNF are supplied to OPCs grown in the presence concentrations of CSPGs, the OPCs are able to overcome the CSPG mediated inhibition, undergoing bipolar process outgrowth and differentiation [176].

In addition to NT treatment, another method for promoting remyelination of spared axons is through the use of antibodies to block the protein Leucine Rich Repeat and Ig Domain Containing 1 (LINGO-1). LINGO-1 is highly inhibitory to the myelination process and is selectively expressed in both oligodendrocytes and neurons. The expression of this protein is developmentally controlled, is known to be upregulated following CNS disease or injury, and inhibits the differentiation and maturation of OPCs via the activation of RhoA pathway [177, 178]. Studies have demonstrated in animal models of demyelination (autoimmune encephalomyelitis or lysolecithin-induced) that utilization of LINGO-1 knockout animals or administration of anti-LINGO-1 antibodies results in significantly increased levels of remyelination [178–180]. With respect to human populations, the use of anti-LINGO-1 as a method of medical treatment for multiple sclerosis (MS) cleared phase I clinical trials in April 2012 and has since moved into phase II [181–183]. While the findings and clinical trials for anti-LINGO-1 antibodies revolve around demyelinating conditions such as MS, anti-LINGO-1 does present another potential therapeutic opportunity for the treatment of SCI.

Promotion of remyelination has also been attempted via cellular transplantation. Transplanting cells, such as OPCs [184–186], Schwann cells (SCs) [187, 188], olfactory ensheathing cells (OECs) [186, 187, 189], or stem cells [186, 190–193], into the site of a demyelinating lesion, has been found to enhance CNS remyelination and subsequent recovery of function. While the results from these studies suggest that cell implantation may be an effective method for remyelinating spared axons, there are some technical difficulties and biological incompatibilities [186, 194] that need to be considered. One such issue is the potential incompatibility between the implanted cell and the endogenous environment of the lesioned CNS. This phenomenon has been noted following transplantation of SCs, which, capable of remyelinating denuded CNS axons, are unable to migrate within CNS tissue or integrate with astrocytes. This unfavorable interaction between SCs and the CNS environment has been documented both* in vitro*, with the failure of SCs to integrate with astrocytes [195], and* in vivo*, with implanted SCs failing to migrate beyond the lesion border [196]. OECs, on the other hand, do integrate with astrocytes [195] but still do not migrate within the damaged spinal cord following injury [197, 198]. Given that transplanted cells are unable to migrate and integrate appropriately, it is not surprising that other problems related to the implantation of stem cells include the possibility of tumorigenicity and the inability to ensure that the stem cells will differentiate into the desired myelin forming cell, as opposed to another phenotype dictated by local environmental influences [185, 194].

Remyelination of spared axons is an enticing avenue of research, given that it may explain an apparent disconnect within the reported findings of many axonal regeneration studies, which have demonstrated paradoxical functional recovery without full anatomical regeneration. While most studies can show evidence of increased axonal sprouting into the spinal cord lesion site, very few studies show that these axons grow beyond the lesion [199]. Thus, functional recovery must be due to some mechanism other than axonal reconnection. This leaves open the possibility that observed recovery in function could be due to the remyelination of spared axons.

## 4. Part III: Use of Biomaterials to Promote Repair

The complex nature of the spinal cord injury dictates that multiple agents will be needed to maximize repair (reviewed by [200]). The lesion itself is usually an irregular size, more often being a partial injury, as opposed to a complete transection. The cellular response, as described in detail above, creates an environment that is not very conductive to repair. Astrogliosis produces a gliotic scar expressing high levels of CSPGs that inhibit axonal regeneration. Neurons that are not connected to their target cells will attempt to regenerate axons and reconnect but are most often unsuccessful. These neurons can survive for several months, but the cell bodies themselves will atrophy and eventually die if connections are not restored [147, 201, 202]. In order to achieve complete restoration of motor function, it is clear that a combination of NTs, to maintain neuronal survival and stimulate axonal regrowth, as well as agents such as cABC, to neutralize the inhibitory effects of the scar, will be required.

One major limitation of many promising treatment strategies for spinal cord injuries is the method of delivery. Most of the therapeutic agents described above have to be delivered via an injection, series of injections, implantation of a pump or intrathecal catheter, use of a viral vector, or implantation of fibroblasts or other cells genetically engineered to produce a given NT or cABC [100, 142, 147, 156, 174, 203–206]. These delivery methods are highly invasive, which could trigger further astrogliotic scarring and inflammation, potentially causing additional neurological damage. Furthermore, the use of viral vectors and implanted genetically engineered cells that deliver a NT or cABC treatment presents an uncontrolled method of delivery and a potential tumor hazard. The use of minipumps and intrathecal catheters provide a nonspecific method of treatment prone to clogging or infection [207]. Thus, utilizing biomaterials that can assist with delivery and control of these agents is a promising area in the field of spinal cord repair.

### 4.1. Critical Issues in the Design of Biomaterials

At the present time, there is no agreement on the optimal characteristics for biomaterials used to repair of the damaged spinal cord [208]. While many different polymers and molecules have been utilized to treat SCI, there are clearly important considerations that cannot be ignored in the development and engineering phase of such biomaterials. First there is the biocompatibility of the material with the host tissue. The developed materials should not elicit an immune response nor be toxic to cells over long periods of time. If the material is biodegradable, the degradation products also should not be toxic to the surrounding tissue [209, 210]. Second, any biomaterial device should be easily introduced into the spinal cord without producing further damage. This can be challenging, as the natural response to spinal injury is the generation of a glial scar. Third, the device must be able to remain in place over long periods of time, in order to allow for nerve growth. This is particularly important for nanoparticles, which are often used for drug delivery. However this consideration is equally necessary for scaffolds and other types of implants. The final critical concern is the ability of the material to bind growth promoting molecules like NT, peptides, and cells, all of which would be delivered in bioactive forms to the injury site to stimulate tissue repair. Scaffolds alone will not be sufficient to enable the maximal repair of the damaged lesions.

The use of biomaterials in the spinal cord generally falls into one of three classes: guidance channels and scaffolds, hydrogels, and nanoparticles. Each one of these can be produced with different chemical compositions, uses, and biological compatibilities. The bioengineering and design of these materials is a very active research field and have been extensively described in the literature [211–214]. Thus, the remainder of this review will be focused on promising applications of biomaterials in the treatment of spinal cord injury.

### 4.2. Guidance Channels and Scaffolds

Guidance channels have been proposed as far back as the late 1800s, with the thought that demineralized bone tubes could be used to fix nerve gaps [215]. The idea behind guidance channels or conduits is to seal the two severed ends of the nerve in a hollow tube, which will direct new axonal regrowth towards the distal nerve stump. Nerve conduits have been successful in repairing peripheral nerve damage, since it is relatively easy to isolate the two individual nerve endings. This is particularly true of larger nerves. However, when considered for use in a spinal cord injury, guidance channels may be better for the transected spinal cord rather than the contusion injury. Unfortunately, the majority of SCIs are incomplete contusion injuries [3], which do not leave discrete nerve stumps and are unable to be sealed effectively using guidance channels. The lesion site in such injuries is irregular and ultimately becomes a cyst-like structure. Therefore, methods of either filling in or crossing these irregularly shaped gaps with a growth promoting substrate are needed. As such, scaffolds that can be inserted into the lesion site are more often being utilized for the contused spinal cord lesion.

In the case of peripheral nerve injury, there are several nerve conduits that are FDA approved for the repair of peripheral nerve gaps that are 30 mm or less (reviewed by [216, 217]). Almost all are made from biodegradable materials that include natural materials like collagen I or synthetic polymers such as polyglycolic acid (PGA) and poly-DL-caprolactone. While they all degrade at slow rates to allow time for the nerve to regenerate, it is imperative that total degradation occurs, ensuring that remaining fragments do not trigger scarring. The current conduits are permeable to nutrients and oxygen and flexible but strong enough to support the nerve and maintain its position during the repair process. While this form of treatment is being utilized in the clinic, there is a clear need for more clinical studies to evaluate the relative efficacy in promoting peripheral nerve repair.

Various forms of scaffolds have been designed to be placed into the spinal cord lesion in order to provide a bridge through the cavitations formed following injury (reviewed by [210]). These provide a permissive and growth promoting environment that allows axons to grow through the lesion unimpeded. Similar to guidance channels, these can be made out of natural materials such a collagen I, agarose, or fibronectin, as well as synthetic polymers like polylactic acid (PLA), PGA, or poly(2-hydroxyethyl methacrylate) (pHEMA). The structure of these scaffolds can vary greatly: they can be cylindrical or rectangular, resemble a multichannel guidance channel, or are sponge-like, with numerous scattered pores. There are also some designs with complex, defined paths which are intended to direct axon growth from specific nerve tracts [218, 219].

Scaffolds and guidance channels can incorporate both growth promoting molecules and a variety of cells that may assist in speeding axon growth through the lesion. The growth factors can be incorporated or attached to the scaffold itself [204, 220]. More often, the scaffold is seeded with cells that produce neurotrophic factors. Schwann cells, genetically altered fibroblasts, or neural stem cells have often been included in various scaffold matrices [221–226]. All scaffold designs are porous structures that are being optimized for long term survival of transplanted cells, while allowing for the infusion of nutrients, oxygen, and formation of new vasculature.

One important feature of any scaffold is that it should not elicit a host reaction to the implant. It was noted early that any implant into the spinal cord that was not biodegradable would activate a tissue response that ultimately resulted in the implant being encapsulated in reactive cells and separated from the host tissue [227]. Activation of immune cells such as macrophages and microglia may alter the effectiveness of any implant. Thus, most scaffolds are biodegradable over time, with their surfaces being modified to manage the host tissue response. This has been accomplished by using materials that encourage the attachment and even infiltration of endogenous cells such as fibroblasts, immune cells, and OPCs that are found close to the lesion site.

The advantage of scaffolds is that they bridge an area of the lesion that is inhospitable with axon regeneration. There are numerous studies that can demonstrate enhanced axonal growth and even some motor improvement, when such techniques are utilized in an experimental model of SCI (reviewed by [210, 228]). Moreover, scaffolds can be designed to guide the direction of new axonal growth through the use of microchannels or other tracts patterned into the scaffold. The disadvantage with this technique is that scaffolds have to be surgically implanted directly into the lesion. Since the dimensions of an injury site tend to be quite irregular, it may be difficult to find an optimal design that will work within each instance of spinal lesion.

### 4.3. Hydrogels

Hydrogels are water saturated polymers that can be developed to mimic the three-dimensional physical properties of the host environment (reviewed by [229, 230]). These polymers can be used in the creation of implantable scaffolds which, as described above, provide a bridge across irregular lesion sites. However, one important quality that makes hydrogels especially appealing for use in SCI repair is that many are able to be injected directly into the lesion site, where they can polymerize* in vivo*. Such polymers are extremely flexible and can fill irregularly shaped lesion cavities by absorbing water, expanding, and forming a flexible three-dimensional structure that closely resembles the extracellular matrix (ECM). Additionally, peptides can be designed to undergo triggered self-assembly, allowing for the formation of hydrogel scaffolds in response to specific changes in the physiological environment (reviewed by [230, 231]). Such hydrogels can also be loaded with growth promoting molecules such as NT or cells that can stimulate axon growth and tissue repair and can even be utilized with a defined scaffold design to maximize growth potential.

Hydrogels can be classified into two general categories: natural and synthetic, referring to the origin of the molecules being used. Mammalian ECM-based natural polymers such as collagen, fibronectin, hyaluronic acid, or combinations are often used in hydrogel creations because of their biocompatibility and the fact that they are part of the naturally occurring ECM. Such substances can be used as a cell-delivery vehicle to promote neurite outgrowth while also providing structural support to the regenerating tissues [206, 232]. Other naturally occurring polysaccharides such as chitosan, agarose, alginate, xyloglucan, gellan gum, and methylcellulose have also shown promise in treating SCI. Often these are used in various combinations, in an attempt to optimize growth promoting properties. All are slowly biodegradable over time, but the rates of degradation are set based on the properties of the molecules and cannot be readily altered.

Synthetic polymers are being developed which can be optimized for maximal protein, cell binding, and rates of degradation. Some of the most common synthetics used for CNS repair have been developed from poly(hydroxyethyl methacrylate) (pHEMA) and derivatives, poly-ethylene-glycol (PEG)/poly-ethylene oxide (PEO), poly(vinyl alcohol) (PVA), and poly(alpha-hydroxyacids). Being synthetic, these substances have some inherent advantages over the natural molecules. They can be manufactured easily, and the properties of the polymers can be customized, to maximize the desired capabilities. For example, the surface of the polymer gel can be optimized for cell attachment. Control of degradation rate can protect or release cells that are transplanted in the hydrogel, depending on the need and the role of these cells in the repair process. Since they are not derived from animals, the potential for allergic reactions to the hydrogel is also minimal [229]. Like scaffolds, both natural and synthetic polymers can be modified to deliver a range of agents, from NT and other growth factors to antagonists for axonal growth inhibitors like Nogo-66 [233–235].

While hydrogels show promise as a potential strategy to maximize repair of the spinal cord, there is no obvious polymer or combination of polymers that are optimal for this application. Therefore the identification of such agents is an active field of research. There are concerns that the mechanical strength of the hydrogel is not sufficient to sustain the lesion and that hydrogels have a shorter durability than fabricated scaffolds because they degrade quickly. More importantly, there is no directionality of the microchannels formed after polymerization* in vivo*. If the hydrogel is rich in growth promoting molecules, regenerating processes could extend into the hydrogel and remain there, mimicking the “honey pot” effect [161]. Overall, specific patterning may be required to actually direct the growth of regenerating axons through the lesion.

### 4.4. Nanoparticles

An alternative approach to treatment of the damaged spinal cord is the use of nanoparticles, which can be used to administer growth factors, NT, and antagonists to inhibitory substances in the lesion. Nanoparticles and microspheres are polymer derived particles that can degrade over time to release any encapsulated agents. These are being widely tested in a myriad of drug delivery applications in multiple tissues, from alleviating tissue rejections from allografts, to targeting cancer cells [236, 237]. These are an attractive delivery system as they are injectable, they provide localized drug delivery without systemic effects, the dose can be titrated easily, and they can target a specific cell type by modification of the cell surface properties. Based on these features, nanoparticles are a major focus of drug delivery methods.

Nanosphere delivery of growth factors and other agents can be successfully used to treat the spinal cord lesion. Drug delivery after an SCI is difficult because of the loss of vascularization and the instability of some of the more promising agents. Chondroitinase ABC is one such agent: it can degrade the glycan residues attached to CSPGs that inhibit axonal growth, neutralizing the effects of these CSPGs. However, it is highly unstable in solution, losing most of its activity within days [106]. It shows great promise in experimental models of spinal cord injury, allowing for substantial growth through a spinal lesion and improved functional recovery [98–100, 103, 238–242]. However, for translation to a human patient population, new delivery methods need to be developed. Nanospheres containing cABC have been developed and utilized in rat spinal contusion models [110]. These nanoparticles can release a sustained supply of active enzyme over the course of three weeks minimum and generate substantial axonal growth through the lesion site. They are nontoxic and do not elicit an inflammatory reaction in the spinal cord. Modulation of the surface charge ensures that they remain in the injury site. They can also be effective in the digestion of CSPGs at chronic times after injury, when the glial scar is fully established.

Nanoparticles and nanospheres that deliver GDNF, BDNF, and NT3 are being developed for several applications in the CNS, including SCI (reviewed by [218, 243, 244]). Other drugs are also being delivered to the spinal cord by nanospheres, including methylprednisolone and estrogen, which are anti-inflammatory agents that can have untoward side effects if administered systemically [245, 246]. Many nanoparticles are fabricated from synthetic polymers such as PLGA and PGA, which can be titrated to regulate the release kinetics. However, they can also be produced using natural polymers such as chitosan. At present, nanoparticles have to be injected into the spinal cord directly; however, recent research efforts are focused on surface modifications that can allow for the nanoparticles to cross the blood-brain-barrier and enter the brain and spinal cord without a direct injection into the tissue. Nonetheless, a direct injection of nanoparticles is much less invasive than the introduction of a scaffold at the lesion.

Nanosphere delivery of therapeutic molecules is attractive for treatment of SCI for many reasons: they are minimally invasive and provide sustained local drug release, which results in a higher dose locally without systemic side effects. Moreover, administration of multiple agents of growth factors could be accomplished by an injection of a mix of nanospheres containing growth factors, cABC, and Nogo antagonists. However, there are many questions that need to be answered prior to use of nanoparticles in a clinical setting. Some of the more critical questions concern the release rates and dose of the agents released from nanoparticles. For example, how much of a particular agent will be required before therapeutic effects occur? Moreover, how long will such agents need to be released into the post-lesion site? cABC and methylprednisolone may be needed acutely, but NT and other molecules may be needed at later stages following injury. A formulation that is released for months has not yet been manufactured in any experimental condition. Nanoparticles could be included in hydrogels to extend release times at the lesion site, if needed [242]. Details on optimal doses and release times in the context of an SCI will need to be determined in order to optimize the use of nanoparticles as a drug delivery system.

## 5. Discussion and Future Directions

Spinal cord injuries are complex and difficult to repair. Research efforts thus far have characterized many molecular events that occur at the injury site, allowing for the identification of several avenues for therapeutic intervention. In short, interventions aimed at promoting functional recovery following spinal cord injury may be targeted towards the neutralization of inhibitory proteoglycans, support of neuronal survival, and stimulation of axonal regeneration and remyelination. The clinical consensus is that there is no single therapeutic agent that can effectively address these issues and that maximal restoration of motor function will most likely be achieved with a mix of agents, each optimized to target a specific aspect of the postinjury response. The other significant problem that persists in developing treatments for SCI relates to the delivery of therapeutic agents to the spinal cord at the appropriate time to facilitate repair. Axonal regeneration is a slow process which, depending on the size of the lesion, could take many months or years in humans. Unfortunately, at present, little is known about how long various therapeutic agents will be needed, their biological half-life and bioactivity* in vivo*, or exactly when they should be introduced into the damaged spinal cord in order to obtain maximal therapeutic effects.

The use of biomaterials provides a promising avenue for addressing the above-mentioned concerns regarding spinal cord repair. Such materials can be utilized not only to deliver therapeutic agents but also to provide physical support for the damaged tissue. Additionally, both natural and synthetic polymers can be used to fabricate several types of structures that can release therapeutic agents with customizable release kinetics. While extensive literature on scaffolds, hydrogels, and nanoparticles exists, there is currently no consensus as to which material is most optimal in repairing the lesioned spinal cord. Ultimately, as new biological requirements of damaged spinal tissue are discovered, the development of biomaterials specialized for the treatment of SCI will need to consider how each of these requirements plays a role in the natural injury response process and potential for functional recovery.
